# RETRACTION: Facial Adult Female Acne in China: An Analysis Based on Artificial Intelligence over One Million

**DOI:** 10.1111/srt.70238

**Published:** 2025-10-08

**RETRACTION**: T.‐H. Li, X.‐D. Ma, Z.‐M. Li, N.‐Z. Yu, J.‐Y. Song, Z.‐T. Ma, H.‐T. Ying, B. Zhou, J.‐Z. Huang, L. Wu, and X. Long, “Facial Adult Female Acne in China: An Analysis Based on Artificial Intelligence Over One Million,” *Skin Research and Technology* 30, no. 4 (2024): e13693, doi:10.1111/srt.13693

The above article, published online on 4 April 2024 in Wiley Online Library (wileyonlinelibrary.com), has been retracted by agreement between *Skin Research and Technology*; and John Wiley & Sons Ltd. The article was accepted for publication following an insufficient peer review process that does not meet the standards of the journal's peer review policy. During post‐publication review, it was identified that the article fails to provide essential information necessary for interpreting and reproducing the findings, and presents irrelevant and/or only marginally relevant citations resulting in it lacking adequate support from the existing literature. Finally, the authors did not disclose the presence of potential conflicts of interest, as several co‐authors are employed by the developer of the smartphone application used in the study. Accordingly, the article has been retracted. The authors have been informed of this decision and disagree with it.

